# Design and Analysis of CFRP Drilling by Electrical Discharge Machining

**DOI:** 10.3390/polym14071340

**Published:** 2022-03-25

**Authors:** Luis Roldan-Jimenez, Fermin Bañon, Ana P. Valerga, Severo R. Fernandez-Vidal

**Affiliations:** Department of Mechanical Engineering and Industrial Design, School of Engineering, University of Cadiz, Av. Universidad de Cadiz 10, E-11519 Puerto Real, Cadiz, Spain; luis.roldan@uca.es (L.R.-J.); fermin.banon@uca.es (F.B.); raul.fernandez@uca.es (S.R.F.-V.)

**Keywords:** surface quality, geometrical, EDM, hole drilling, electrode, kerf, composites, capacitance, tolerance

## Abstract

The novelty of EDM machining as a drilling operation for composite materials means that there is no consensus on the influence of the parameters that optimise the final quality. For these reasons, a characterisation of the EDM drilling process of a unidirectional composite material has been developed. The influence of several cutting parameters has been related to the quality of the final hole obtained. Thus, macrogeometric aspects in terms of conicity and final diameter and microgeometric aspects in terms of surface quality have been evaluated. In addition, the final state of the material and the wear of the electrode have been evaluated by visual inspection, allowing the range of cutting parameters that offer the best performance to be established. Finally, a series of contour diagrams based on predictive models have been obtained to establish a direct relationship between input and output variables.

## 1. Introduction

Carbon fibre reinforced thermosetting composites have generated a great deal of interest in the last decade due to their excellent ratio of mechanical properties to light weight [1]. This has allowed a potential increase in applications oriented to the aeronautical or automotive sector or sectors such as electronics [2,3,4].

However, its anisotropic composition favours the use of specific machining technologies to comply with the geometric tolerances established in the industry. In particular, drilling operations, whose tolerances are of great relevance in the aeronautical sector, stand out. Consequently, numerous studies have been carried out in the aeronautical sector focusing on the optimization of conventional drilling of composite materials by evaluating temperatures and tolerances [5], studying tool wear [6,7], using cryogenic cooling [8] or focusing on the machining of these materials in the form of hybrid structures [9,10].

Specific cutting geometries are required which can increase the final cost in order to achieve small tolerances. In addition, the abrasive nature of the reinforcement and adhesive wear constantly deteriorate the cutting edges resulting in large variations in the final diameter [9]. Alternatives such as the application of lubricants or cryogenic coolants can improve the final process performance, but at the same time increase the negative environmental impact or alter the composition of the composite material [11,12,13].

Therefore, non-conventional machining processes can be an alternative in order to improve the final performance of the process and minimize its environmental impact. Processes such as abrasive water jet machining or laser machining are alternatives that are currently being widely studied [14,15,16]. Nevertheless, defects associated with both processes hinder the accuracy of the drilling process of composite materials [17,18]. The dissipation of kinetic energy of the water jet results in a lag of the final machining zone with respect to the initial zone [19]. This generates considerable variations in the final diameter due to the taper generated.

On the alternative, the nature of the laser machining process results in high machining temperatures that can burn or deteriorate the thermoset matrix generating a thermally affected zone [20]. In addition, EDM die-sinking process can be a high quality and precision drilling process compared to other machining processes [21,22].

The characteristics of EDM machining enable a controlled process. Cutting tools with a very wide range of diameters allow large diameter holes to be drilled or to focus on micro drilling processes [23,24]. At the same time, it is a process characterized by minimising final geometric deviations and achieving a high final surface quality.

Nonetheless, the EDM machining of composite materials presents a great number of challenges today. As there is no electrical conductivity between the matrix and electrode, the dielectric properties of the matrix must be overcome for fibre-to-fibre discharge to occur [25]. This is removed and adheres to the electrode, which can lead to variations in the final diameter [26]. At the same time, machining temperatures can lead to die loss during machining, resulting in a poor surface finish and deterioration of the workpiece [27].

In addition, part of the electrode is removed during the process producing a continuous variation that causes a taper in the hole drilled. Some research works focused on the drilling of thermoset composite materials by EDM are shown in Table 1. Current studies are mainly based on micro EDM (μEDM) drilling operations with tungsten carbide, graphite or copper rotary tools, and analyze the surface quality of the drilling, deviations, delamination and matrix adhering to the electrode and the workpiece.

In the field of CFRP drilling by EDM, Kumaran et al. [26] have studied the machining of an epoxy matrix unidirectional CFRP. For this purpose, they used a 1 mm diameter brass electrode. A surface modification was carried out by sanding to remove the first layer of resin in order to facilitate the conductivity in the initial stages. In their research, they have studied the influence of the pause time (20–42 µs), the current intensity (46–153 A) with a fixed value for the 250 V voltage. They have established a relationship between these parameters and the rate of material removal, electrode wear and final hole quality.

Regarding the influence of cutting parameters, Dutta et al. [29] have developed an experimental design on 1.4 mm thickness CFRP microdrilling. Voltage (100–190 V), discharge time (10–40 µs) and electrode rotation speed (200–500 rpm) have been modified at different levels. The authors emphasize that the most influential parameter on the final quality is the voltage. In addition, they establish the material removal mechanisms for this process. They state that the electrode particles after machining remain adhered to the inner walls of the hole. On the other hand, the combination of electrode wear and the sparks produced in improperly machined carbon filaments leads to an increase in bore taper.

In another study, Dutta et al. [34] focuses on machining microchannels by optimizing cutting parameters. In their study, they highlight the mechanism of polymer matrix removal through a detachment phenomenon. This arises from the thermal stress generated which produces micro-cracks in both the reinforcement and the matrix leading to subsequent debonding. In addition, Dutta et al. report that after the first discharges a pyrolytic carbon layer is produced which increases the conductivity of the subsequent chips.

Kumar et al. [35] have developed an experimental study focusing on CFRP microdrilling with 120 µm diameter electrodes. In their study they focus on carbon fibre layers by varying the voltage (80–120 V), capacitance (33–1000 Pf) and electrode rotational speed (1000–2000 rpm). Their study highlights the relationship between voltage and capacitance through a parameter called “discharge energy”. They establish that high values of discharge energy increase the rate of material removal as well as electrode wear [33]. Conversely, lower values of this parameter result in more controlled machining, improving the surface quality of the holes.

Due to the novelty of EDM machining as a drilling operation for composite materials, there is no consensus on the influence of the parameters that optimize the final quality. For these reasons, a characterization of the EDM drilling process of a unidirectional composite material has been developed. The influence of several cutting parameters has been related to the quality of the final hole obtained. Thus, macrogeometric aspects in terms of taper and final diameter and microgeometric aspects in terms of surface quality have been evaluated.

In addition, the final state of the material and the electrode wear has been evaluated by visual inspection allowing to establish the range of cutting parameters that offer the best performance. Finally, a series of contour diagrams based on predictive models have been obtained in order to establish a direct relationship between input and output variables.

## 2. Materials and Methods

**Figure 1:**
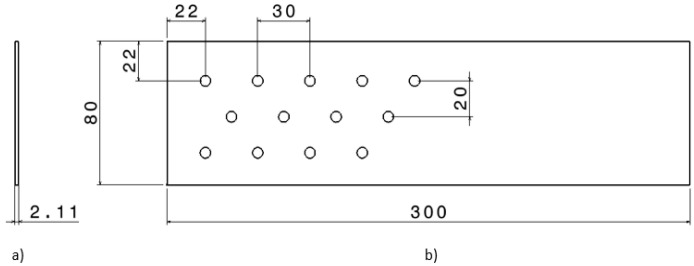
Measurements of the workpiece (mm) and test arrangement. (**a**) Side view. (**b**) Top view.

An ONA NX3 EDM machine (Ona, Durango, Spain) was used to carry out the study. The copper electrode has a cylindrical geometry of 6 mm diameter and 40 mm height. The workpiece is made of carbon fibre with an epoxy matrix and has a rectangular geometry shown in Figure 1.

**Figure 2:**
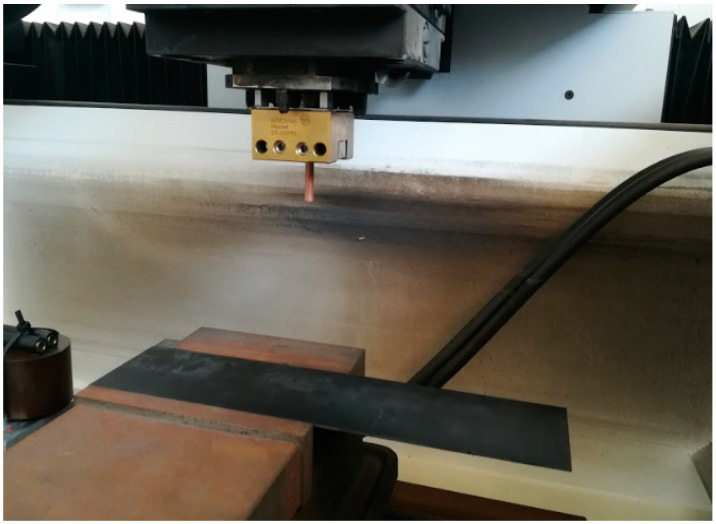
Tool holder positioned in the central column.

It should be noted that the arrangement of the carbon fibres is in one direction only, i.e., it is a unidirectional continuous carbon fibre workpiece. The specimens have been laminated with a 34% resin ratio, giving a laminate density of 1.58 g/cm^3^. Table 2 shows the physicochemical properties of the materials used in the study. The placement of the electrode and the unidirectional composite material on the equipment is shown in the Figure 2.

The factors and technological values that have the greatest influence on the process according to the bibliography studied have been selected as input variables for the process. Table 3 below shows the selected factors and their different levels. Following these same criteria, the rest of the factors necessary for the development of the process listed in Table 4 have been established.

In addition, a Taguchi design with orthogonal array L9(3)4, shown in Table 5, was used to carry out the experimentation. Four additional trials have been developed, trials 10 and 11 are replicates of trial 3 and trials 12 and 13 are replicates of trial 8. These trials have been chosen to demonstrate the robustness of the design.

Once the different experiments have been carried out, images are taken with a stereo optical microscope (SOM) Nikon® SMZ800 (Nikon Inc., Chiyoda, Japan) at the entrance and exit of the hole. The Material Removal Rate (MRR), as well as the Gap and the Kerf are calculated. The Kerf parameter has been evaluated as the difference in diameter between the upper and lower diameters after machining.

In addition, a contact profilometer, Mahr Perthometer PGK 120 (Mahr GmbH, Göttingen, Germany), was used for microgeometric characterization. Measurements of Ra (μm) and measurements of Rz (μm) are taken from four generatrices of the hole and a distinction is made between areas in which machining occurs perpendicular to the direction of the carbon fibre (zone 0–180°) and areas in which machining occurs parallel to the direction of the carbon fibre (zone 90–270°).

## 3. Results

### 3.1. Surface Quality

**Figure 3:**
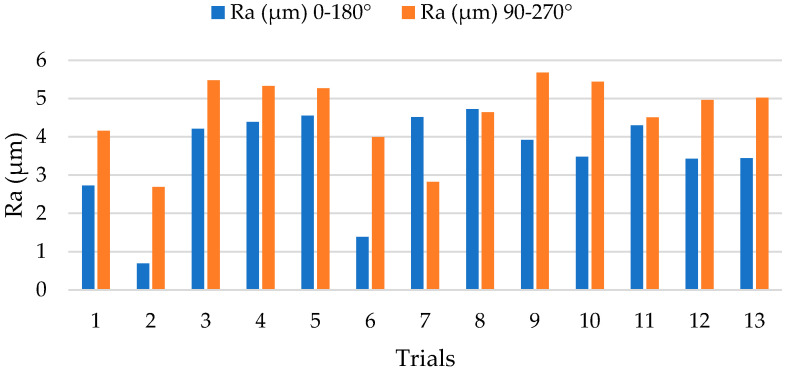
Surface roughness values after EDM drilling tests in two evaluation orientations.

The results obtained for the surface quality in terms of Ra are shown in Figure 3. These have been evaluated in orientations parallel (0–180°) and perpendicular (90–270°) to the orientation of the carbon fibre reinforcement.

High Ra values have been obtained in a 90–270° orientation due to the electrical conductivity of the reinforcement. Due to the anisotropy of composite materials, the heat propagation generated during machining occurs in the orientation of the reinforcement (0–180°). This allows for a better controlled electrical discharge in this orientation and an increase in the machining temperature. This increase enhances the loss of thermoset matrix by vaporization.

In contrast, the propagation between parallel reinforcements is lower due to the lack of physical continuity (90–270°). Due to the fact that the heat generated in the process is mainly transmitted in the orientation of the reinforcement, the volume of matrix evaporated by the temperatures can be smaller. This makes the loss of polymer matrix more difficult and leads to greater irregularity at the periphery of the bore and a poorer surface finish.

On the contrary, a more stable process is observed with very close values for most of the tests carried out in the 0–180° orientation. In particular, tests 2 and 6 stand out with Ra values below 2 µm. This would indicate that the drilling of unidirectional composite materials by EDM can generate final surface qualities within the tolerances of the aeronautical sector.

**Figure 4:**
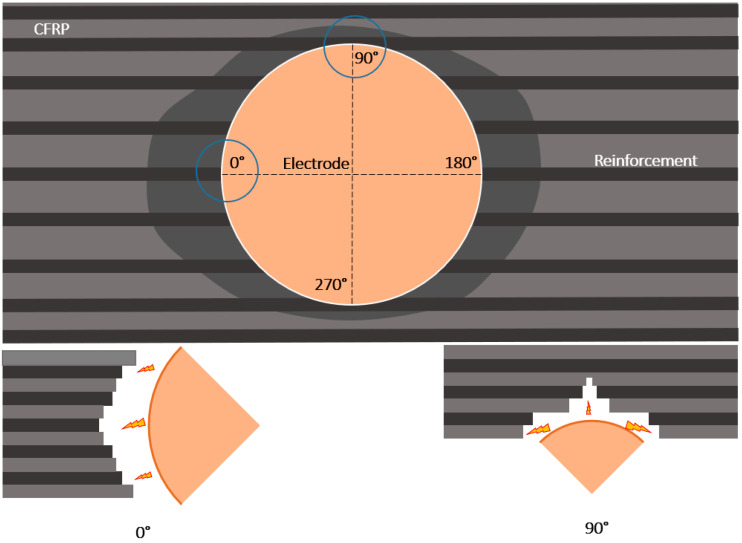
Graphical representation of electric discharge propagation as a function of the fibre orientation.

Thus, the influence of the cutting parameters on the surface quality in the 0–180° orientation is shown in Figure 4. The direct statistical influence of all the parameters established in the experimental design is highlighted.

**Figure 5:**
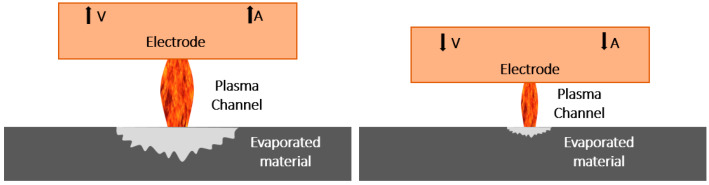
Graphical representation of the influence of machining parameters on the amount of evaporated material.

However, of all the modified parameters, voltage has the greatest influence on the final surface quality, followed by intensity, capacitance level and pulse time, respectively. Therefore, it can be indicated that the surface quality will depend on the correct combination of voltage and intensity values. Parameters that directly affect the intensity of the discharge generated. Higher values of these parameters give a higher material removal capacity to the process. This produces higher temperatures that evaporate the thermoset matrix more easily, but leads to deeper craters, resulting in poorer final surface quality (Figure 5).

In comparison, for the 90–270° orientation the surface quality depends directly on the variation in discharge intensity and set capacitance as the most statistically significant parameters (Table 6 and Table 7). DF is the total degrees of freedom; Adj MS indicates the mean squares measure and how much variation a term or a model explains and Adj SS is the adjusted sums of squares are measures of variation for different components of the model. Additionally, the *p*-value is a probability that measures the evidence against the null hypothesis. Lower probabilities provide stronger evidence against the null hypothesis and higher values of F indicate a high influence on the response.

**Figure 6:**
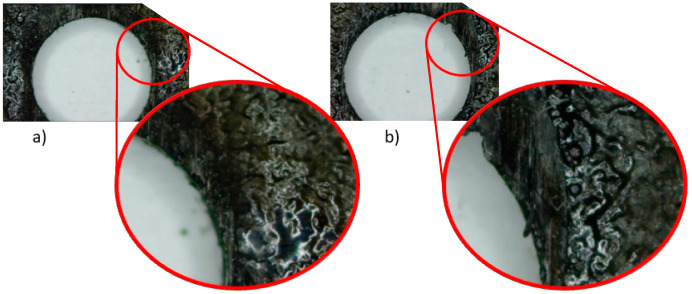
Thermal defects observed in the matrix after drilling tests. (**a**) Capacitance level 25. (**b**) Capacitance level 35.

This can be seen in the individual trends obtained in Figure 6. An increase in intensity produces a higher machinability to the process generating a rougher and more random surface. This is essential for the 90–270° orientation due to the thermal properties of the reinforcement, higher intensities are required to correctly machine the matrix and improve the quality. However, very high intensity values (8 A) can be excessive and burn the matrix leaving the composite material unmachined and generating an unstable gap that deteriorates the final surface quality.

The direct relationship between the surface quality in terms of Ra and the voltage set is also clear. Therefore, in order to obtain a better surface quality, understanding better surface quality as reduced Ra values, voltage values close to 80 V must be set.

In addition, the roughness varies considerably depending on the pulse time set. Values as low as 100 µs would indicate that the time in which the electrical discharge acts on the material is insufficient. In particular, if an inadequate time is not set, the temperatures reached during the process may be insufficient, making it difficult to machine the die and resulting in a variation in the roughness generated [26].

Furthermore, there is a tipping point for a time of 150 µs where a correct relationship between the electrical discharge time and the channel cleaning time is established, resulting in a smoother and more controlled surface.

Finally, a pulse time of 200 µs can lead to an unstable process in which the intervals between each electrical discharge are very short. This can result in the cleaning of the evaporated material not being carried out correctly, destabilising the process [25]. This is important as the main mechanism of matrix removal is through evaporation by the process temperatures. If not correctly evacuated, these can adhere to the electrode and to the machined surface itself, altering the final surface quality.

It has also been observed that an increase in capacitance allows more energy to be stored during the process, improving the stabilization of the process. This generates a constant material removal improving the final surface quality [25,33].

**Figure 7:**
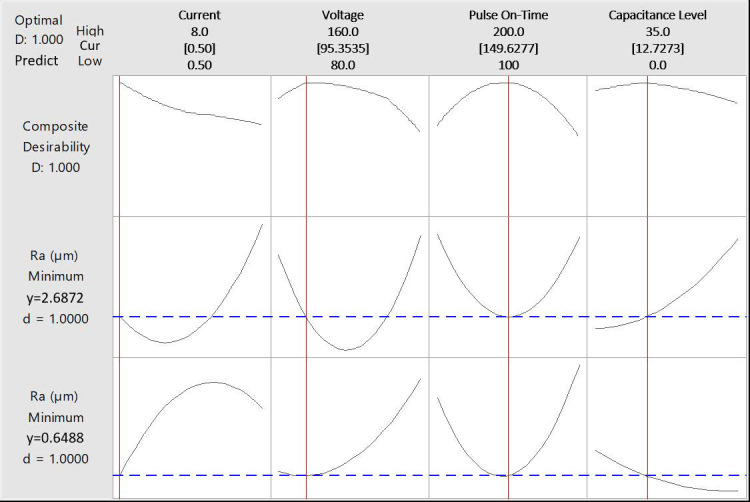
Combination of machining parameters reducing Ra in both reinforcement orientations.

So, in order to establish a stable process, a combination of a discharge current of 0.5 A, a voltage of 95 V, a pause time of 150 µs and a capacitance of 13 F can produce the lowest Ra values in both directions (Figure 7).

**Figure 8:**
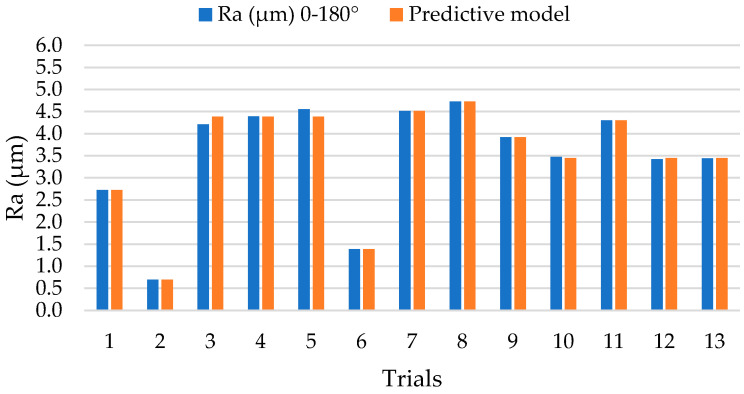
Comparison of Ra values between experimental tests and theoretical values of the predictive model for an orientation 0–180°.

**Figure 9:**
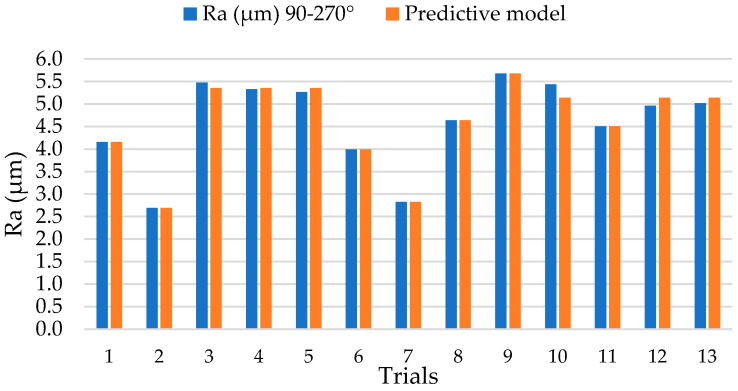
Comparison of Ra values between experimental tests and theoretical values of the predictive model for an orientation 90–270°.

From the results, a predictive model has been obtained that relates the process variables with the surface quality obtained in both directions of evaluation. Thus, for the 0–180° orientation, the model (1) has been obtained with a fit of 99.67% and for the 90–270° orientation, the model (2) has been obtained with a fit of 98.59%. This would indicate a high degree of reliability where the maximum errors committed are 4% and 5%, respectively, in comparison with the experimentally obtained results (Figure 8 and Figure 9).

Ra (µm) [0–180°] = 20.01 + 0.8163 I − 0.0808 V_s_ − 0.2127 t_on_ − 0.0473 C − 0.07584 I^2^ + 0.000430 V_s_^2^ + 0.000730 t_on_^2^ + 0.000721 C^2^ (1)

Ra (µm) [90–270°] = 20.70 − 0.295 I − 0.1728 V_s_ − 0.1089 t_on_ − 0.0005 C + 0.0510 I^2^ + 0.000732 V_s_^2^ + 0.000362 t_on_^2^ + 0.000829 C^2^, (2)

where Current (I), Voltage (V_s_), Impulse time (t_on_), and Capacitance (C).

**Figure 10:**
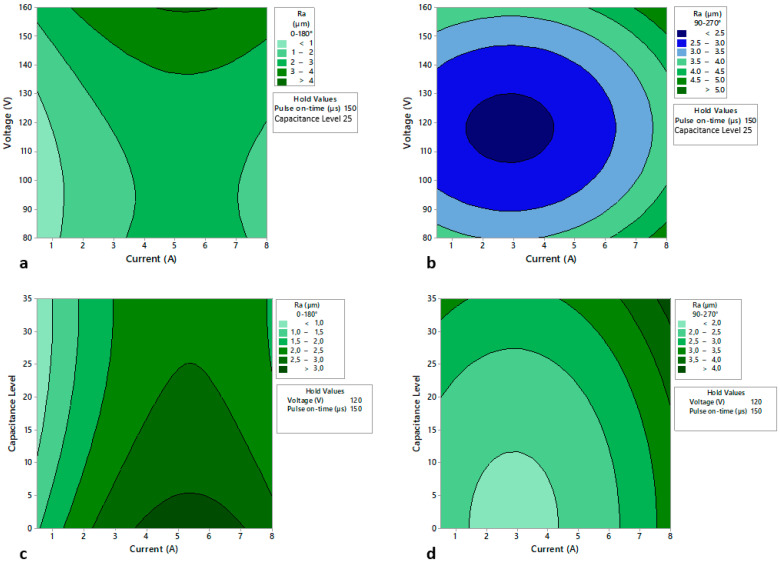
Contour diagrams that relate the surface quality in terms of Ra to the following machining parameters (**a**) Voltage and current for the 0–180° orientation; (**b**) Voltage and current for the 90–270° orientation; (**c**) Capacitance and current for the 0–180° orientation; (**d**) Capacitance and current for the 90–270° orientation.

In addition, through the predictive model, a series of contour diagrams have been established that relate the surface quality in both directions of evaluation with the most significant parameters of the process (Figure 10).

Y. Akematsu et al. [27] studied the erosion behaviour, with the help of an infrared thermal camera, of the copper electrode on carbon fibre composite material. This study shows that the electrical discharge moves in the longitudinal direction of the carbon fibres, causing better finishes in the area where it is machined perpendicular to the direction of the carbon fibre, in this report, this area is 0–180°. The same can be applied to the Ra 90–270° studies, where the carbon fibre is parallel to the machining, and to the Rz studies. It also shows that capacitance is the factor with the greatest effect on machining for this type of material, which is in agreement with the results presented.

In addition, Y. H. Guu et al. [36] studies the EDM machining of copper electrode on carbon fibre composite material, obtaining as a result that, when the capacitance factor does not intervene in the machining, the roughness parameter maintains the same trend as the one obtained in this report, increasing as the pulse time and the current intensity increase.

### 3.2. Material Remove Rate

One of the main limitations in EDM machining is the volume of material removed per unit time. Due to the characteristics of the process, material removal rates are reduced [37].

**Figure 11:**
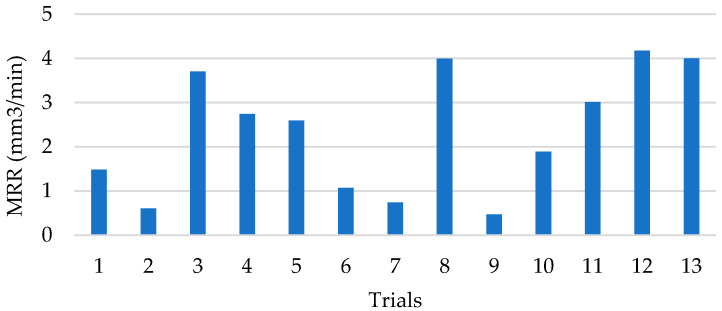
Measured material removal rate values for the developed tests.

As a result, machining parameters that minimize the operating time are required. The values of material removal rates in EDM drilling of composite material are shown in (Figure 11).

There is a large variability of results depending on the combination of cutting parameters. Nonetheless, it can be seen that the highest removal rates are achieved with maximum capacitance parameter in the set range. In this aspect, the energy discharged during each process pulse is directly related to the capacitance and the set voltage values [25,32]. Increasing the capacitance value increases the energy that can be stored and subsequently discharged. This increase in discharged energy generates a larger volume of vaporized material for a unit of time, increasing the productivity of the machining process [34]. This is corroborated in the ANOVA analysis in (Table 8). The capacitance parameter is the only significant parameter in the material removal rate study. So, an increase in the volume of material requires high capacitance values that allow an increase in the energy discharged and minimize the resistance of the material to be machined.

The anisotropy of the composite material, however, makes it difficult to machine using this technology compared to monolithic elements such as metal alloys [38]. This is reflected in the repeatability of the results obtained. Thus, the removal rate values obtained in tests 3, 10 and 11 vary from 3.7 to 2 mm^3^/min. This may be due to the volume of polymer matrix during machining. The matrix removal depends on the temperature reached [29,32].

However, if there is a higher percentage of matrix or if the matrix is not properly vaporized during machining, it can hinder the electrical conductivity between the reinforcement. This can lead to a dulling of the electrode, coated with polymer matrix and increased difficulty in machining. This in combination with a reduced current (0.5 A) can enhance such variation in results.

**Figure 12:**
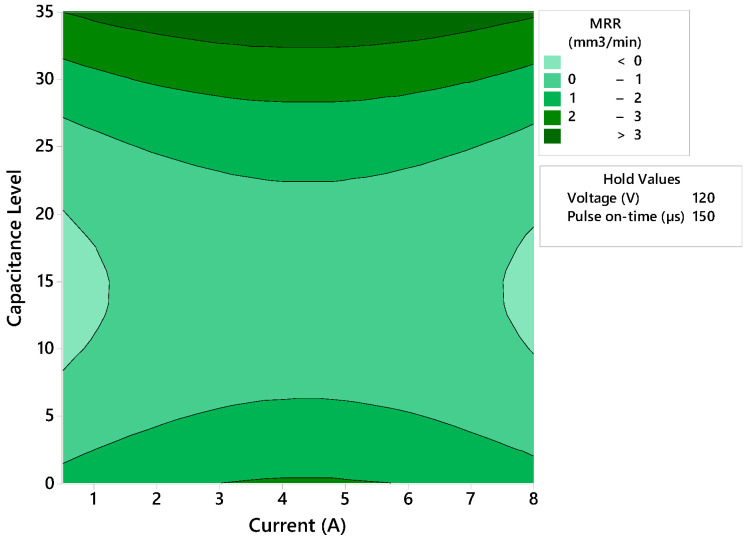
Contour diagram that relates the material removal rate to the two main machining parameters.

A predictive model with a 92.65% fit has been obtained from the experimental results (Figure 12). This has made it possible to obtain a contour diagram showing the relationship between capacitance and intensity established for each combination of cutting parameters and to establish a relationship with the results obtained for surface quality.

In order to increase productivity, high capacitance values are necessary for all set intensity levels. This allows removal rates of more than 3 mm^3^-min to be reached. Higher capacitance values store more energy which is subsequently discharged. This minimizes the resistance of the composite material, allowing higher machining temperatures to be reached that vaporize the polymer matrix and removing a greater amount of material per electrical discharge. Notably, lower current levels are required to achieve maximum material removal rates in EDM drilling of composite materials compared to micro EDM drilling operations [26].

### 3.3. Kerf

To obtain this Kerf output variable, the diameters of the holes in the upper part are measured, in addition to the diameters of the holes in the lower part, with these measurements the difference between the two can be calculated and the dimensional deviation can be obtained as a result. The output bore is smaller than the input bore, and the aim is to minimize the value obtained, because this minimizes the dimensional deviation between the upper and lower bore of the workpiece.

**Figure 13:**
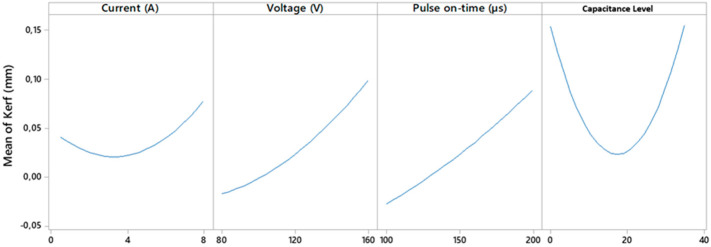
Individual influence of machining parameters on taper results.

Figure 13 shows the trends of the individual parameters. The voltage and pulse time parameters seem to have a clear influence with the deviations of the input and output diameters of the holes made, while the current or capacitance parameters do not have such a clear influence.

It is observed that the coefficient of the stress factor and the coefficient of the impulse time factor are statistically significant for the response Table 9. Furthermore, the standard error of the coefficients is low, indicating that the model has good repeatability.

**Figure 14:**
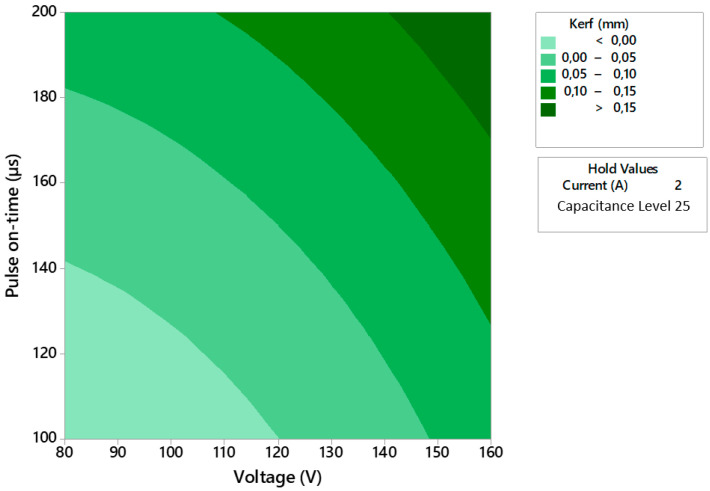
Contour diagram that relates the taper defect to the two main machining parameters.

A predictive model (3) with an R2 of 96.75% has been obtained from the experimental results. This has made it possible to obtain a contour plot showing the relationship between stress and pulse time for each combination of shear parameters and to establish a relationship with the results obtained for the Kerf (Figure 14).

Kerf (mm) = 0.060 − 0.0171 I − 0.00120 V_s_ + 0.00023 t_on_ − 0.01489 C + 0.00259 I^2^ + 0.000011 V_s_^2^ − 0.000003 t_on_^2^ + 0.000427 C^2^ (3)

where Current (I), Voltage (V_s_), Impulse time (t_on_), and Capacitance (C).

R. Abdallah et al. [39] studies the influence of the factors on the Kerf parameter, machining unidirectional carbon fibre composite material using WEDM, obtaining that the most influential factors are the current intensity and the pause time, and that this parameter decreases as the current intensity increases. However, this report shows that the factors that have the greatest effect on the Kerf parameter are voltage and pulse time, with the Kerf parameter increasing as either of these factors increases.

Finally, as a result of the study of the effects on the Kerf parameter data, the proposed solution to optimize this output parameter. Minimum taper values are obtained for a current of 3 A, a voltage of 80 V, a pulse time of 100 µs, and a capacitance level of 17.5.

## 4. Conclusions

The control factors in die sinking EDM technology with copper electrodes applied to CFRP with unidirectional fibres for the development of holes have been analysed.

The surface quality showed significant differences in the measured orientations, parallel (0–180°) and perpendicular (90–270°), due to the anisotropy of the material. The current and intensity values were found to be significant for the measurements made in the 0–180° orientations with a minimum Ra value of 0.69 µm and 2.69 in the 90–270° orientation. It is concluded that higher values of these parameters produce larger discharges that produce larger craters, resulting in a worse surface quality.

Capacitance turns out to be the most influential factor in the material removal rate. The study has shown that, in order to optimize this response results, high values of capacitance should be used. This is because, as the capacitance value increases, the amount of energy provided for machining increases, thus removing more material.

The increase in energy results in an increase in the temperature of the affected area which favours the evaporation of the material matrix, affecting the quality of the results.

The dimensional deviation between the hole entry and exit is directly related to the stress factors and the pulse time. This study shows that, in order to improve the value of this parameter, low values for both voltage and pulse time should be used. This parameter is actually a process defect, since it is the dimensional deviation caused between the hole entry and exit, caused by the energy not being transmitted correctly over the entire electrode surface when the electrode has penetrated to the defined depth in the workpiece.

## Figures and Tables

**Table 1:** Machining parameters used in the consulted bibliography.

Ref.	Voltage (V)	Amperage (A)	Pulse Time (µs)	Tool Speed (rpm)	Capacitance (µF)	Electrode
[28]	50–70	2–6	70–90	-	-	Cu
[29]	100–190	-	10–40	200–500	-	Cu
[30]	45	2–8	600	-	-	Cu, W
[25]	80–120	-	-	1000–2000	33–1000	W
[31]	65	0.4–2	20–190	-	-	Cu, C
[27]	200–500	-	-	-	1–100	Cu
[32]	80–110	5–15	0.6–20		-	Cu
[33]	80–100	-	-	1000–2000	0–100	W

**Table 2:** Physicochemical properties of the materials used.

Properties	Copper	Epoxy Matrix Carbon Fibre	
		Graphite	Epoxy
Density (g/cm^3^)	8.9–8.94	1.63–1.67	1.11–1.40
Electrical resistivity (μΩ × cm)	1.4–5.01	158–501	1022
Melting point (°C)	982–1080	3.53 × 10^3^–3.68 × 10^3^	66.9–167
Specific heat capacity (J/kg × °C)	372–388	852–941	4.08 × 10^5^–5.5 × 10^5^

**Table 3:** Selected factors and experimental levels.

Level	Current (A)	Voltage (V)	Impulse Time (μs)	Capacitance Level
1	0.5	80	100	0
2	1	120	150	25
3	8	160	200	35

**Table 4:** Fixed factors and selected values.

Factor	Value
Pause time (μs)	50
Working time (s)	0.75
Reverse time (s)	0.25
Servo (V)	45
Electrode polarity	Positive
Security level	3
Criteria	Minimal wear

**Table 5:** Selected factors and experimental levels.

Trials	Current (A)	Voltage (V)	Impulse Time (μs)	Capacitance Level
1	0.5	80	100	0
2	0.5	120	150	25
3	0.5	160	200	35
4	1	80	150	35
5	1	120	200	0
6	1	160	100	25
7	8	80	200	25
8	8	120	100	35
9	8	160	150	0

**Table 6:** Statistical analysis (ANOVA) of the influence of the machining parameters on the surface quality for 0–180° orientation.

Source	DF	Adj SS	Adj MS	F-Value	*p*-Value
Model	8	18.6421	2.33027	153.14	0
Current (A)	1	3.1955	3.19553	210	0
Voltage (V)	1	5.4271	5.42707	356.65	0
Impulse time (µs)	1	0.7927	0.79269	52.09	0.002
Capacitance level	1	1.1567	1.15669	76.01	0.001
Error	4	0.0609	0.01522		
Total	12	18.703			

**Table 7:** Statistical analysis (ANOVA) of the influence of machining parameters on surface quality for 90–270° orientation.

Source	DF	Adj SS	Adj MS	F-Value	*p*-Value
Model	8	11.1388	1.39235	35.02	0.002
Current (A)	1	2.0815	2.08149	52.36	0.002
Voltage (V)	1	0.085	0.08501	2.14	0.217
Impulse time (µs)	1	0.0013	0.00126	0.03	0.867
Capacitance level	1	1.9264	1.92643	48.46	0.002
Error	4	0.159	0.03975		
Total	12	11.2978			

**Table 8:** Statistical analysis (ANOVA) of the influence of machining parameters on material removal rate.

Source	DF	Adj SS	Adj MS	F-Value	*p*-Value
Model	8	21.3109	2.66386	6.3	0.047
Current (A)	1	0.0201	0.02012	0.05	0.838
Voltage (V)	1	0.0597	0.05967	0.14	0.726
Impulse time (µs)	1	0.0346	0.03458	0.08	0.789
Capacitance level	1	5.6093	5.60926	13.26	0.022
Error	4	1.6918	0.42296		
Total	12	23.0027			

**Table 9:** Statistical analysis (ANOVA) of the influence of machining parameters on taper defect.

Source	DF	Adj SS	Adj MS	F-Value	*p*-Value
Model	8	0.108133	0.013517	14.87	0.010
Current (A)	1	0.002580	0.002580	2.84	0.167
Voltage (V)	1	0.022661	0.022661	24.94	0.008
Impulse time (µs)	1	0.025933	0.025933	28.54	0.006
Capacitance level	1	0.000005	0.000005	0.01	0.946
Error	4	0.003635	0.000909		
Total	12	0.111769			
